# Management of Atrial Fibrillation in Patients on Ibrutinib: A Cleveland Clinic Experience

**DOI:** 10.7759/cureus.2701

**Published:** 2018-05-29

**Authors:** Sidra Khalid, Samin Yasar, Aariez Khalid, Timothy PP Spiro, Abdo Haddad, Hamed Daw

**Affiliations:** 1 Internal Medicine Residency, Fairview Hospital, Cleveland Clinic, Cleveland, USA; 2 Internal Medicine, Fairview Hospital, Cleveland Clinic, Cleveland, USA; 3 Biomedical Science, University of Guelph, Guelph, CAN; 4 Department of Hematology and Oncology, Fairview Hospital, Cleveland Clinic, Cleveland, USA

**Keywords:** chronic lymphocytic leukemia, waldenstrom’s macroglobulinemia, mantle cell lymphoma, atrial fibrillation, ibrutinib, rate control, rhythm control, anticoagulation

## Abstract

Background

Ibrutinib is a Bruton’s tyrosine kinase inhibitor, which is United States Food and Drug Administration (FDA)-approved for chronic lymphocytic leukemia, mantle cell lymphoma, and Waldenström’s macroglobulinemia. Ibrutinib is associated with atrial fibrillation and bleeding events. Our aim is to determine the management of prior atrial fibrillation when starting ibrutinib, as well as ibrutinib-induced atrial fibrillation. Our focus is on which rate and rhythm control strategies to use and decisions regarding the use of antiplatelet and anticoagulation agents.

Materials and Methods

We conducted a retrospective descriptive study of case records over a three-year period from February 2014 to February 2017. We reviewed 597 patient charts from the Cleveland Clinic database. Ibrutinib was started in 43 patients. Of those, 10 had atrial fibrillation prior to starting ibrutinib and four developed atrial fibrillation while on ibrutinib. Data was collected for demographic details, co-morbid conditions, CHA_2_DS_2_-VASc (congestive heart failure, hypertension, age, diabetes mellitus, prior stroke, transient ischemic attack or thromboembolism, vascular disease, age, and sex category) score, HAS-BLED (hypertension, abnormal renal and liver function, stroke, bleeding, labile INR, elderly, and drugs or alcohol) score, and drugs used for antiplatelet effects, for anticoagulation, and for rate and rhythm control. Outcomes for embolic and bleeding events were assessed.

Results

Of the 43 patients, 14 (32.5%) had or developed atrial fibrillation; 10 (23.26%) had prior atrial fibrillation, and four (9.30%) developed atrial fibrillation after starting ibrutinib. The majority were males (71.42%) and Caucasian (71.42%). The disease breakdown was chronic lymphocytic leukemia (42.86%), mantle cell lymphoma (50%), and Waldenström’s macroglobulinemia (7.14%). The mean starting dose of ibrutinib in patients with prior atrial fibrillation was 569 mg and for patients who developed atrial fibrillation was 420 mg. In the 10 patients who had atrial fibrillation prior to ibrutinib, all 10  were on beta blockers, one was on diltiazem, three were on amiodarone, one was on flecainide, one was on digoxin, and one was on Tikosyn® (Pfizer, Inc., New York, NY). The ibrutinib dose was decreased/discontinued in two patients. In patients who developed atrial fibrillation after starting ibrutinib, three were on beta blockers, two on amiodarone, and one on Tikosyn. Ibrutinib was discontinued in one patient. In patients who had prior atrial fibrillation, three were on warfarin, one on enoxaparin, and two on apixaban. In three patients, aspirin and enoxaparin were discontinued. In patients who developed atrial fibrillation after starting ibrutinib, enoxaparin was given to two and apixaban to one. None of the patients had a stroke, transient ischemic attack (TIA), or bleeding events.

Conclusions

From our study, we concluded that ibrutinib can be safely given in the presence of atrial fibrillation, and when atrial fibrillation was induced, we further concluded that beta blockers were the preferred agents for rate control. Ibrutinib has many drug interactions with other rate and rhythm control agents; hence, their use was lower. When atrial fibrillation was uncontrolled, ibrutinib was temporarily held and then cautiously restarted. The decision to start or adjust anticoagulation depended on the bleeding and stroke risks as assessed by their physicians.

## Introduction

Ibrutinib is an oral Bruton’s tyrosine kinase inhibitor, which has been approved by the United States Food and Drug Administration (US FDA) for the treatment of chronic lymphocytic leukemia, mantle cell lymphoma, and Waldenström’s macroglobulinemia [1]. Ibrutinib has two adverse effects: atrial fibrillation and bleeding. The management of atrial fibrillation is challenging in patients who require anticoagulation. In patients with atrial fibrillation, rate and rhythm control is imperative. However, some of the drugs available for rate and rhythm control can alter the concentration of ibrutinib, leading to changes in the efficacy and/or the adverse effects profile of ibrutinib. Additionally, as ibrutinib can cause bleeding, it is crucial to assess the bleeding risk in these patients. Our study aimed to identify patients with prior atrial fibrillation treated with ibrutinib (and with new-onset ibrutinib-related atrial fibrillation) to determine the appropriate management of atrial fibrillation with respect to rate and rhythm control and anticoagulation, as well as to determine bleeding risks. We also assessed the complications of atrial fibrillation by examining the patients’ CHA_2_DS_2_-VASc (congestive heart failure, hypertension, age, diabetes mellitus, prior stroke, transient ischemic attack or thromboembolism, vascular disease, age, and sex category) and HAS-BLED (hypertension, abnormal renal and liver function, stroke, bleeding, labile INR, elderly, and drugs or alcohol) scores. Outcomes assessed included embolic and bleeding events in these patients.

## Materials and methods

We conducted a retrospective descriptive chart review study over a three-year period from February 2014 to February 2017. We reviewed 597 charts from the Cleveland Clinic database. A total of 43 patients received ibrutinib. Of these 43 patients, 10 had atrial fibrillation prior to starting ibrutinib and four developed atrial fibrillation after starting ibrutinib. Inclusion criteria included patients above the age of 18 years with either chronic lymphocytic leukemia, mantle cell lymphoma, or Waldenström’s macroglobulinemia. Data variables that were collected included demographic details, co-morbid conditions, CHA2DS2-VASc score, HAS-BLED score, starting dose of ibrutinib, changes in dose or discontinuation of ibrutinib, rate and rhythm controlling agents used, antiplatelet/anticoagulation agents used, other management options for atrial fibrillation, and outcomes – stroke, embolic events and bleeding. Bleeding was characterized as “major” (intracranial, intraspinal, intraocular, retroperitoneal, intraarticular or pericardial, or intramuscular with compartment syndrome) and “minor”.

## Results

A total of 43 patients were started on ibrutinib. Of these 43 patients, 14 (32.5%) had or developed atrial fibrillation. Out of the 14 patients, 10 (23.26%) had atrial fibrillation prior to starting ibrutinib and four (9.30%) developed atrial fibrillation after starting ibrutinib. A total of 15 patients had chronic lymphocytic leukemia; five of them had pre-existing atrial fibrillation, and one developed atrial fibrillation. Mantle cell lymphoma was diagnosed in 22 patients; four of them had pre-existing atrial fibrillation, and three developed atrial fibrillation. Waldenström’s macroglobulinemia was diagnosed in six patients, out of which one patient had pre-existing atrial fibrillation, and none developed atrial fibrillation (Table 1).

**Table 1:** Distribution of Patients on Ibrutinib According to Malignancy and Atrial Fibrillation

Malignancy	# of patients	Started on ibrutinib	Had atrial fibrillation prior to starting ibrutinib	Developed atrial fibrillation after ibrutinib was started
Chronic lymphocytic leukemia	325	15	5	1
Waldenström ’s macroglobulinemia	70	6	1	0
Mantle cell lymphoma	202	22	4	3
TOTAL	597	43	10 (23.26%)	4 (9.30%)

The majority of our patients were male (71.42%) and Caucasian (71.42%). Relevant co-morbid conditions included hypertension 78.57%, diabetes mellitus 21.43%, and congestive heart failure 21.43%. The mean starting dose of ibrutinib in patients with prior atrial fibrillation was 569 mg and for patients who developed atrial fibrillation was 420 mg. In patients who developed new-onset atrial fibrillation related to ibrutinib, the atrial fibrillation occurred in all four patients within one year of starting ibrutinib (Table 2).

**Table 2:** Baseline Patient Characteristics afib: atrial fibrillation; TIA: transient ischemic attack; DM: diabetes mellitus; HTN: hypertension; CAD: coronary artery disease; CHF: congestive heart failure

	All -14	Atrial fibrillation prior to starting ibrutinib (10)	Atrial fibrillation after starting ibrutinib (4)
Gender, male	10 (71.42%)	7 (70%)	3 (75%)
Race			
Caucasian	10 (71.42%)	8 (80%)	2 (50%)
Black	2 (14.29%)	1 (10%)	1 (25%)
Other	2 (14.29%)	1 (10%)	1 (25%)
Malignancy			
Chronic lymphocytic leukemia	6 (42.86%)	5 (50%)	1 (25%)
Mantle cell lymphoma	7 (50%)	4 (40%)	3 (75%)
Waldenström’s macroglobulinemia	1 (7.14%)	1 (10%)	0
Atrial fibrillation	14	10	4
Starting dose of ibrutinib – mean	430 mg	569 mg	420 mg, onset of afib within a year: 4 (100%)
Co-morbid conditions			
Age (mean) at time of atrial fibrillation and ibrutinib	68.23	65.77	73.75
Prior history of stroke or TIA	0	0	0
Vascular disease	0	0	0
DM	3 (21.43%)	1 (10%)	2 (50%)
HTN	11 (78.57%)	7 (70%)	4 (100%)
CAD	6 42.86%)	6 (60%)	0
CHF	3 (21.43%)	3 (30%)	0
Renal dysfunction	3 (21.43%)	2 (20%)	1 (25%)
Thyrotoxicosis	0	0	0
Alcohol abuse	0	0	0
Smoking	5 (35.71%)	4 (40%)	1 (25%)
Chronic liver disease	0	0	0
Chronic lung disease	3 (21.43%)	2 (20%)	1 (25%)
Substance abuse	0	0	0

Rate and rhythm controlling agents

Of the 10 patients who had atrial fibrillation prior to ibrutinib, the rate control and anti-arrhythmic agents used were beta blockers - 10, diltiazem - 1, amiodarone - 3, flecainide - 1, digoxin - 1, and Tikosyn® (Pfizer, Inc., New York, NY) - 1. After starting ibrutinib, eight patients had changes made to their rate control and anti-arrhythmic agents. The beta blockers were stopped or the dose was reduced in six patients, the beta blocker dose was increased in one, and the diltiazem dose was decreased in one. No dose adjustments for amiodarone, flecainide, digoxin, or Tikosyn were made. The ibrutinib dose was decreased or discontinued in two patients. One patient required an ablation procedure, and three underwent cardioversion.

In patients who developed atrial fibrillation after starting ibrutinib, the rate control and anti-arrhythmic agents used were beta blockers - 3, amiodarone - 2, and Tikosyn - 1. For one patient, amiodarone and Tikosyn were stopped and also ibrutinib was discontinued (Table 3).

**Table 3:** Rate and Rhythm Controlling Agents Used and Changed with Ibrutinib

Rate and rhythm controlling agents:			
	All - 14	Atrial fibrillation prior to starting ibrutinib (10)	Atrial fibrillation after starting ibrutinib (4)
Beta blockers (BB)	13 (92.86%)	10 (100%)	3 (75%)
Diltiazem	1 (7.14%)	1 (10%)	0
Amiodarone	5 (35.71%)	3 (30%)	2 (50%)
Flecainide	1 (7.14%)	1 (10%)	0
Digoxin	1 (7.14%)	1 (10%)	0
Tikosyn	2 (14.29%)	1 (10%)	1 (25%)
Change in rate control drugs – discontinued/decreased dose/changed to a different drug	9 (64.29%)	8 (80%) (doses of beta blockers reduced or stopped x6, Cardizem decreased x1, beta blockers increased x1)	1 (25%) (stopped amiodarone and Tikosyn)
Change in dose of ibrutinib or discontinued	3 (21.43%)	2 (20%)	1 (25%)
Cardioversion (yes)	3 (21.3%)	3 (30%)	0
Ablation (yes)	1 (7.14%)	1 (10%)	0
Watchman device (yes)	0	0	0

Anticoagulation

In patients who had atrial fibrillation and were started on ibrutinib, the CHA2DS2-VASc scores were 1 for one patient, 2 for five patients, and 3 for three patients. HAS-BLED scores were 1 for two patients, 2 for seven patients, and 3 for one patient. The antiplatelet and anticoagulant agents used were aspirin - 9, warfarin - 3, enoxaparin - 1, and apixaban - 2. In three patients, aspirin and enoxaparin were discontinued. None of the patients suffered a stroke, TIA, or any bleeding events. One patient had a systemic embolism.

In patients who developed atrial fibrillation after starting ibrutinib, CHA2DS2-VASc scores were 3 for two patients and 4 for two patients. HAS-BLED scores were 1 for one patient, 2 for two patients, and 3 for one patient. The antiplatelet and anticoagulation agents used were aspirin - 3, enoxaparin - 2, and apixaban - 1. The dose of apixaban was reduced in one patient (25%). For outcomes, no patient had a stroke, transient ischemic attack (TIA), systemic embolism, or bleeding events (Table 4).

**Table 4:** Anticoagulation and Outcomes of Atrial Fibrillation with Ibrutinib CHA_2_DS_2_-VASc (congestive heart failure, hypertension, age, diabetes mellitus, prior stroke, transient ischemic attack or thromboembolism, vascular disease, age, and sex category); HAS-BLED (hypertension, abnormal renal and liver function, stroke, bleeding, labile international normalized ratio, elderly, and drugs or alcohol); TIA: transient ischemic attack

Antiplatelet agent or anticoagulants			
	All -14	Atrial fibrillation prior to starting ibrutinib (10)	Atrial fibrillation after starting ibrutinib (4)
Aspirin	12 (85.71%)	9 (90%)	3 (75%)
Warfarin	3 (21.43%)	3 (30%)	0
Enoxaparin	3 (21.43%)	1 (10%)	2 (50%)
Apixaban	3 (21.43%)	2 (20%)	1 (25%)
Changes in antiplatelet or anticoagulation agents	4 (28.57%)	3 (30%) (discontinued enoxaparin; discontinued aspirin; discontinued aspirin)	1 (25%) apixaban dose reduced
CHA_2_DS_2_-VASc score	% Out of 13		
0	0	0	0
1	1 (7.69%)	1 (11.11%)	0
2	5 (38.46%)	5 (55.56%)	0
3	5 (38.43%)	3 (33.33%)	2 (50%)
4	2 (15.38%)	0	2 (50%)
HAS-BLED score	% Out of 14		
1	3 (21.43%)	2 (20%)	1 (25%)
2	8 (57.14%)	7 (70%)	1 (25%)
3	3 (21.43%)	1 (10%)	2 (50%)
Outcomes	% Out of 14		
Stroke (hemorrhagic, ischemic, TIA)	0	0	0
Systemic embolism	1 (7.14%)	1 (10%)	0
Bleeding (major [intracranial, intraspinal, intraocular, retroperitoneal, intra-articular or pericardial, or intramuscular with compartment syndrome]; minor)	0	0	0

## Discussion

In our study of 43 patients who were started on ibrutinib, four patients developed ibrutinib-related atrial fibrillation within a year of starting the drug. This accounted for about 9.3% of the patients. According to various clinical studies, ibrutinib-related atrial fibrillation ranged from 3.6% to 16% of cases [2-4]. The proposed mechanism of atrial fibrillation caused by ibrutinib is related to the inhibition of Bruton's tyrosine kinase (BTK) and related kinases. BTK regulates the phosphoinositide 3-kinase (PI3K)-protein kinase B (Akt) pathway, which protects the cardiac muscle in stressful conditions. In a study by McMullen et al., neonatal rats’ ventricular myocytes were given ibrutinib and its effect was observed under basal conditions and in the presence of an insulin-like growth factor. In the myocytes with the insulin-like growth factor, ibrutinib caused reduced PI3K-Akt activity. This helps to support the concept that when ibrutinib inhibits the PI3K-Akt pathway, the protective effect is lost and the heart is prone to atrial fibrillation [2].

In our study, in the 10 patients who had atrial fibrillation prior to ibrutinib, all were continued on beta blockers. In the four patients who developed atrial fibrillation after starting ibrutinib, three were started on beta blockers, two on amiodarone, and one on Tikosyn. In one patient, amiodarone and Tikosyn were discontinued and ibrutinib was also discontinued due to the difficulty in controlling the atrial fibrillation. It should be noted that ibrutinib has drug interactions with rate and rhythm controlling agents used for the management of atrial fibrillation. Ibrutinib is metabolized by cytochrome 3A4/5 and drugs, such as diltiazem and verapamil, which inhibit cytochrome 3A4 and increase the concentration of ibrutinib. Ibrutinib also inhibits p-glycoprotein, and it can increase the intracellular concentration of p-glycoprotein substrates, such as digoxin [5]. Amiodarone also inhibits cytochrome 3A4 and can cause a rise in the serum concentration of ibrutinib [6].

In our study, ibrutinib was discontinued or the dose was reduced in three out of 14 patients. Clinical experience has shown that ibrutinib can be temporarily held and then reintroduced at either a full or reduced dose if the atrial fibrillation is controlled [7]. Discontinuation is not usually recommended.

Ibrutinib’s bleeding risk is due to its inhibition of glycoprotein VI and glycoprotein 1b-mediated platelet function. This leads to decreased platelet adhesion to Von Willebrand factor [3]. Due to ibrutinib’s predisposition to cause bleeding, an algorithm was proposed by Vrontikis et al. in which they indicated that bleeding and stroke risk should be assessed with the CHA_2_DS_2_-VASc and HAS-BLED scores. If the stroke risk is more than the bleeding risk, the patient should be anticoagulated. If the bleeding risk is elevated, then anticoagulated or antiplatelet therapy is not indicated, and further evaluation should be performed by a cardiologist [3]. In our study, for patients who had atrial fibrillation prior to starting ibrutinib, anticoagulants were discontinued in three patients after assessing the CHA_2_DS_2_-VASc and HAS-BLED scores. In patients who developed ibrutinib-related atrial fibrillation, three were started on anticoagulants - enoxaparin or apixaban.

For patients with atrial fibrillation, the current cardiology guidelines do not recommend antiplatelet agents for stroke prevention. The European Society of Cardiology recommends warfarin or non-Vitamin K oral anticoagulants (NOACs) for CHA_2_DS_2_-VASc scores of 1 or more. However, with ibrutinib, there is a concern for bleeding. The decision to anticoagulate with warfarin or a NOAC is important. With warfarin, the international normalized ratio (INR) could be monitored and controlled more closely. With NOACs, there is no reversal agent available, except for idarucizumab for dabigatran. Also, in large clinical trials, reduced dose NOACs, dabigatran, and apixaban showed comparable results to warfarin and full-dose NOACs for stroke prevention. Therefore, NOACs at reduced doses or warfarin are treatment options for stroke prevention for patients on ibrutinib [5].

The findings of this study should be interpreted in light of some potential limitations. The main limitation of our study is that the sample size is small. In addition to that, it was a retrospective study, and there was no long-term follow-up on complications, such as bleeding and stroke. This study cannot assess the types of anticoagulants that should be used as no comparison was done between Vitamin K antagonists and NOACs.

## Conclusions

This retrospective descriptive study allowed us to recognize the challenges associated with ibrutinib in the management of atrial fibrillation. For rate control, our study concluded that the use of beta-blockers is preferred, which is similar to other studies, as it carries a low risk of interacting with ibrutinib. In terms of other rate and rhythm control agents, since ibrutinib has many drug interactions, it is recommended to hold ibrutinib temporarily and then resume it at a lower or full dose. Due to the small sample size of our study, we were unable to determine whether interventions, such as cardioversion or ablation, are helpful for patients with atrial fibrillation on ibrutinib. Also, the main issue that was made relevant to the study was the use of anticoagulation and antiplatelet agents for stroke prevention. As ibrutinib has elevated bleeding risk, the CHA_2_DS_2_-VASc and HAS-BLED scores need to be utilized in order to make a well-informed decision. The options for anticoagulation range from warfarin to a low/full dose of NOACs. Further studies are required to determine which form of anticoagulation would be best suited for patients with atrial fibrillation taking ibrutinib. Long-term follow-up is also needed to determine the outcomes of stroke, bleeding and embolic events, as our period of study may have been too short to detect these adverse events.

## References

[1] (2017). FDA Approval for Ibrutinib. Retrieved July 02.

[2] McMullen JR, Boey EJH, Ooi JYY (2014). Ibrutinib increases the risk of atrial fibrillation, potentially through inhibition of cardiac PI3K-Akt signaling. Blood.

[3] Vrontikis A, Carey J, Gilreath JA (2016). Proposed algorithm for managing ibrutinib-related atrial fibrillation. Oncology (Williston Park).

[4] Wiczer TE, Levine LB, Brumbaugh J (2017). Cumulative incidence, risk factors, and management of atrial fibrillation in patients receiving ibrutinib. Blood Adv.

[5] Mulligan SP, Ward CM, Whalley D, Hilmer SN (2016). Atrial fibrillation, anticoagulant stroke prophylaxis and bleeding risk with ibrutinib therapy for chronic lymphocytic leukaemia and lymphoproliferative disorders. Br J Haematol.

[6] (2017). Ibrutinib - Drug Summary. http://www.pdr.net/drug-summary/Imbruvica-ibrutinib-3399.

[7] Thorp BC, Badoux X (2018). Atrial fibrillation as a complication of ibrutinib therapy: clinical features and challenges of management. Leuk Lymphoma.

